# Probiotic NVP-1703 Alleviates Allergic Rhinitis by Inducing IL-10 Expression: A Four-week Clinical Trial

**DOI:** 10.3390/nu12051427

**Published:** 2020-05-15

**Authors:** Min-Gyu Kang, Seung-Won Han, Hye-Ryun Kang, Seok-Jin Hong, Dong-Hyun Kim, Jeong-Hee Choi

**Affiliations:** 1 Department of Allergy and Clinical Immunology, Chungbuk National University Hospital, Cheongju 28644, Korea; irreversibly@gmail.com; 2 Department of Internal Medicine, Chungbuk National University College of Medicine, Cheongju 28644, Korea; 3 PB Business Department, Navipharm Inc., Suwon 16209, Korea; swhan@navipharm.co.kr; 4 Department of Internal Medicine, Division of Allergy and Clinical Immunology, Seoul National University Hospital, Seoul 03080, Korea; helenmed@hanmail.net; 5 Department of Otorhinolaryngology—Head and Neck Surgery, Hallym University Dongtan Sacred Heart Hospital, Hwaseong 18450, Korea; enthsj@hallym.or.kr; 6 Allergy and Clinical Immunology Research Center, Hallym University College of Medicine, Chuncheon 24252, Korea; 7 Neurobiota Research Center, College of Pharmacy, Kyung Hee University, Seoul 02453, Korea; dhkim@khu.ac.kr; 8 Department of Pulmonology and Allergy, Hallym University Dongtan Sacred Heart Hospital, Hwaseong 18450, Korea

**Keywords:** allergic rhinitis, probiotics, immunoglobulin E, interleukin-10

## Abstract

Although several recent studies reported that probiotics might be beneficial for allergic rhinitis (AR), the effect of probiotics on AR is not consistent and have not been reproduced between studies. We aimed to determine the efficacy and safety of probiotic NVP-1703, a mixture of *Bifidobacterium longum* and *Lactobacillus plantarum*, in subjects with perennial AR. Adult subjects with perennial AR received either NVP-1703 (*n* = 47) or placebo (*n* = 48) for four weeks. Total nasal symptom scores (TNSS), rhinitis control assessment test (RCAT), blood eosinophil count, allergen-specific IgE, and immunological parameters in serum and urine were compared at baseline and after four weeks. TNSS changes from baseline at weeks 1, 3, and 4 were significant between the NVP-1703 and placebo groups (*p* = 0.033, 0.031, and 0.029, respectively). RCAT score showed significant differences between the NVP-1703 and placebo groups (*p* = 0.049) at week 4. *Dermatophagoides farinae*-specific IgE levels and serum IL-10 levels were significantly different between the NVP-1703 and placebo groups (*p* = 0.033 and *p* = 0.047, respectively). IL-10/IL-4 and IL-10/IL-13 ratios were different between the NVP-1703 and placebo groups at week 4 (*p* = 0.046 and 0.018, respectively). NVP-1703 treatment reduced urinary prostaglandin F_2α_ and leukotriene E_4_ levels (*p* > 0.05). Therefore, NVP-1703 can be treatment option for perennial AR.

## 1. Introduction

Allergic rhinitis (AR) is one of the most common allergic diseases globally [1]. Typical symptoms include nasal itching, sneezing, rhinorrhea, nasal congestion, and/or red, itchy, and watery eyes, which last throughout the year in perennial AR [1]. Immunoglobin E (IgE)-mediated allergic inflammation is the main pathophysiological mechanism of AR and drives T helper 2 (Th2) cell polarized immune reactions [2]. Th2 cells produce interleukin (IL)-4, IL-5, and IL-13, which activate various effector cells involved in allergic inflammation, such as eosinophils and mast cells [1,2]. Th2-derived allergic inflammation is attributed to a functional defect of T regulatory (Treg) cells, which results in decreased production of IL-10 and transforming growth factor (TGF)-β [3,4]. As IL-10 and TGF- β antagonize the biologic function of Th2-cytokines, decreased IL-10 and TGF-β is considered to subsequently potentiate allergic inflammation [5].

Conventional pharmacological treatments of AR include antihistamines, nasal corticosteroids, and leukotriene receptor antagonists, which are effective in controlling symptoms and allergic inflammation [1]. Allergen-specific immunotherapy is a potential disease-modifying, immune-modulatory treatment option that can activate allergen-specific Treg cells and subsequent production of IL-10 and TGF-β [5]. Although allergen immunotherapy is effective in treating AR, it has some limitations, as some patients experienced moderate to severe adverse events during immunotherapy. 

Previous clinical trials have reported the effectiveness of probiotics in controlling symptoms and improving quality of life in patients with AR [6,7,8,9]. However, the study protocols of outcome measurements have been mixed: some probiotics improved rhinitis quality of life questionnaire scores in several studies, while they had no effect on rhinitis total symptom scores or symptom medication scores in others [10]. Furthermore, the mechanism of anti-allergic activity of probiotics has been investigated with peripheral blood mononuclear cells (PBMCs) from patients with AR. *Bifidobacterium lactis* NCC2818 [6], *Lactobacillus gasseri* A5 [11], *Lactobacillus paracasei* ST11 [12], and *Lactobacillus casei* Shirota [13] reduced IL-5, IL-13, and tumor necrosis factor (TNF)-α production from PBMCs of patients with AR, but did not affect serum total IgE, serum allergen-specific IgE, or IL-10 production. In a randomized, open-label crossover study of *Lactobacillus johnsonii* EM1 in 7–12-year-old children with perennial AR, treatment with EM1 and levocetirizine combined significantly increased serum TGF-β levels compared with levocetirizine alone at week 12 [14]. We previously reported that the noble probiotic NVP-1703, a mixture of *Bifidobacterium longum* IM55 and *Lactobacillus plantarum* IM76, mitigated ovalbumin (OVA)- and house dust mite allergen (HDMA)-induced AR in mice, reducing IL-4, IL-5, IL-13, and IgE levels in nasal mucosa, bronchial alveolar lavage fluid, and blood and increasing the level of IL-10 [15,16], suggesting that probiotics may suppress Th2 response by promoting Treg activity. 

We conducted a clinical trial to assess the efficacy and safety of NVP-1703 for treatment of perennial AR by evaluating total nasal symptom scores (TNSS) and rhinitis control assessment test (RCAT). We also investigated the effect of NVP-1703 on blood eosinophil count, allergen-specific IgE, immunological parameters (IL-4, IL-5, IL-10, IL-13, and INF-γ), and leukotriene (LT)E_4_ and prostaglandin (PG)F_2α_ in serum or urine. 

## 2. Materials and Methods 

### 2.1. Subjects

A total of 95 adult subjects (19–65 years old) with perennial AR were recruited from Hallym University Dongtan Sacred Heart Hospital and Chungbuk National University Hospital. The inclusion criteria were: (1) persistent rhinitis symptoms for at least two consecutive years; (2) presence of two or more rhinitis symptom domains (nasal itching, sneezing, rhinorrhea, and nasal congestion) with ≥2 scores without taking medication; and (3) at least one positive response to skin prick tests for five common perennial allergens (*Dermatophagoides pteronyssinus* (Dp), *Dermatophagoides farinae* (Df), cat, dog, and cockroach). The subjects did not take any drugs or functional foods to relieve nasal symptoms during the run-in period. Additionally, subjects who could not discontinue drugs that might affect AR symptoms during the study period, who received systemic steroids or nasal cromolyn within two weeks or antihistamines within three days of the screening, or who were taking medicine for gastritis, gastric ulcer, or asthma were excluded. Subjects who were taking rhinitis medication were excluded from the study.

All subjects provided written informed consent. The study protocol was approved by the Institutional Ethics Committee of Hallym University Dongtan Sacred Heart Hospital (IRB file no. HDT 2018–01–007) and Chungbuk National University Hospital (CBNUH 2018–01–004). The study was registered at https://cris.nih.go.kr under the identifier KCT0003760.

### 2.2. Study Products

NVP-1703 is a probiotic mixture of *B. longum* IM55 and *L. plantarum* IM76 freeze-dried with maltodextrin. The placebo was made of maltodextrin and manufactured to be indistinguishable in appearance from the NVP-1703 stick pack. According to the instruction of Navipharm Inc. (Suwon, Korea), all study products were produced at a Good Manufacturing Practice-certified manufacturing facility. Both NVP-1703 and placebo were added to 0.5% (*w*/*w*) strawberry concentrate powder and 0.5% (*w*/*w*) strawberry incense cotton to equalize taste and aroma, and were stored at 2–8 °C. 

### 2.3. Study Design

**Figure 1:**
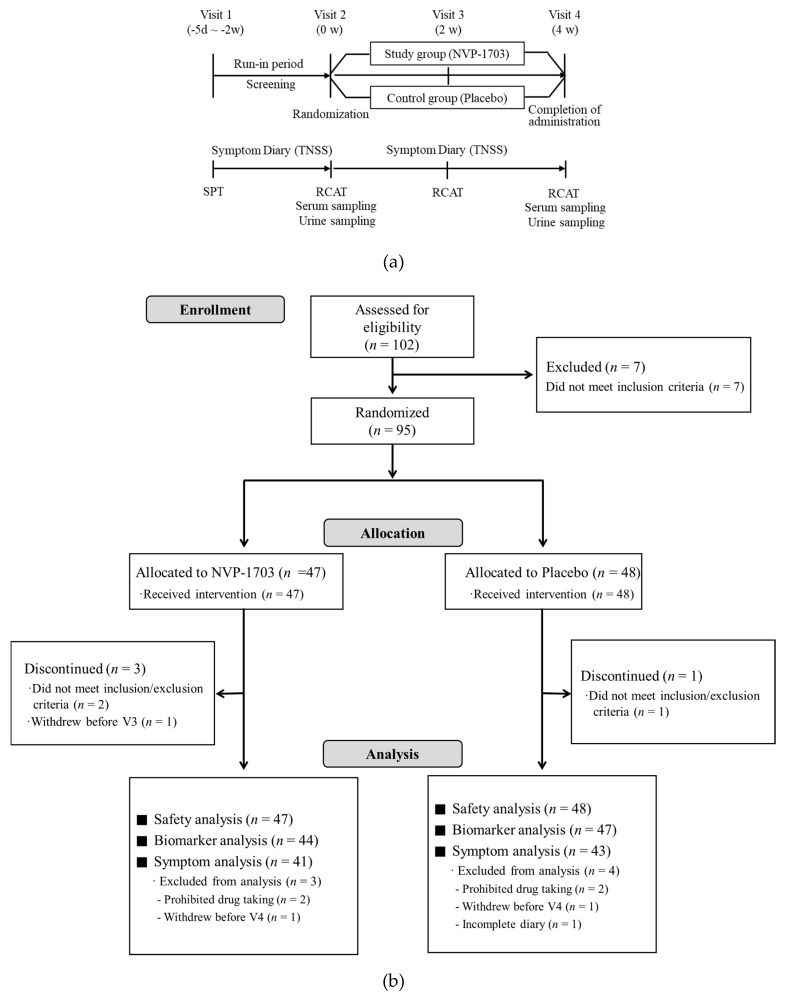
Description of (**a**) study protocol and (**b**) flow diagram of subject disposition. TNSS, total nasal symptom score; SPT, skin prick tests; RCAT, rhinitis control assessment test.

The study was conducted as a multi-center, double-blind, randomized, placebo-controlled clinical trial. The study subjects made a total of four visits during the study (Figure 1). At Visit 1 (screening), demographics, medical history, and medication history were obtained for each participant. Physical examination, laboratory tests, electrocardiogram, and skin prick tests were also performed. After completing a nasal symptom diary during the run-in period of at least 5 days after Visit 1, subjects who met the inclusion criteria were enrolled and assigned to one of the two groups by random numbers generated by the SAS system on Visit 2 (week 0). The randomization ratio for each group was 1:1. NVP-1703 [1.0 × 10^10^ CFU/day (2 g/stick pack)] or placebo (maltodextrin 2g/stick pack) was taken orally once a day for 4 weeks. After Visit 2, the subjects kept daily nasal symptom diaries while taking the investigational products. Visits 3 (week 2) and 4 (week 4) occurred at 2-week intervals after Visit 2. RCAT scores were assessed at Visit 2 and Visits 3 and 4. Peripheral blood eosinophil counts, serum Dp- or Df-specific IgE, IL-4, IL-5, IL-10, IL-13, interferon (IFN)-γ, and urinary LTE_4_ and PGF_2α_ were evaluated at Visit 2 and Visit 4. The primary outcome was the difference in change in TNSS between groups before and after the ingestion of NVP-1703, and secondary outcomes were changes in RCAT scores, immunological parameters, blood eosinophil count, and Dp- or Df-specific IgE.

### 2.4. TNSS

TNSS were expressed as the sum of the scores for the four symptoms (nasal congestion, rhinorrhea, nasal itching, and sneezing). Each symptom was rated on a 4-point scale from 0 (none) through 1 (mild), 2 (moderate), and 3 (severe), and recorded daily in the diary by the subjects during the study. The baseline was the average of the symptom scores recorded during the run-in period, and weekly changes in the symptom scores were analyzed.

### 2.5. RCAT

RCAT, a tool for assessing the level of rhinitis control, consists of six items: nasal congestion, sneezing, watery eyes, sleep interference, daily activities, and degree of rhinitis control. Each item is assigned 1 to 5 points, and the scores of all items are summed up for evaluation. A higher score indicates better rhinitis control. The subjects were surveyed on Visits 2, 3, and 4, and the RCAT score was evaluated at each visit.

### 2.6. Skin Prick Test

Allergy skin prick tests were performed with 12 common inhalant allergens, including Dp, Df, mold mixture 1 (*Alternaria alternata, Chaetomium globosum, Cladosporium, Helminthosporium sativum*), mold mixture 2 (*Aspergillus fumigatus, Aspergillus niger*), cat, dog, cockroach, tree pollen mixture, birch, grass pollen mixture, ragweed, and mugwort (Allergopharma, Hamburg, Germany or Hollister-Stier, Spokane, WA, USA). Skin prick tests were considered positive if the mean wheal diameters were 3 mm or greater. 

### 2.7. Measurement of Inflammatory Mediators in Serum and Urine

Serum Dp- and Df-specific IgE levels were measured by fluorescent enzyme immunoassay using the ImmunoCAP^®^ system (Thermo Fisher Scientific, Uppsala, Sweden). IL-4, IL-5, IL-10, IL-13, and IFN-γ levels were measured in serum using a Human High Sensitivity T-Cell Magnetic Bead Panel kit (Millipore, Billerica, MA, USA) and Luminex LX200 (Millipore). Urinary LTE_4_ and PGF_2α_ levels were measured using a commercial ELISA kit (Cayman Chemical, Ann Arbor, MI, USA) and expressed as ng/mmol creatinine. Creatinine level was measured using a CREJ2 kit with Cobas 8000 (Roche Diagnostics, Mannheim, Germany). 

### 2.8. Statistical Analysis

The normality of each group was calculated using the Kolmogorov–Smirnov test. A two-sample *t*-test was used if the normality was approximated after log transformation of parameters that were not distributed normally. For continuous data among the baseline characteristics, two-sample *t*-tests were performed to compare between groups, and categorical data were analyzed using chi-square or Fisher’s exact test. A two-sample *t*-test was used to compare weekly changes in the TNSS and individual symptom scores between groups, and baseline-adjusted ANCOVA was used for the changes in RCAT scores, blood eosinophil count, serum specific IgE, and serum cytokines and urinary LTE_4_ and PGF_2α_. Cytokine levels were analyzed after log transformation. Changes in the ratios of IL-10 to IFN-γ, IL-4, IL-5, and IL-13 were analyzed by baseline-adjusted ANCOVA. For each item of data, a paired *t*-test was employed within groups. All values were calculated as mean ± standard error of the mean (SEM). Statistical analysis was performed using SAS 9.4 software (SAS Institute, Cary, NC, USA), and *p* values ˂ 0.05 were considered statistically significant. 

## 3. Results

### 3.1. Study Subject Characteristics

Of the 95 subjects, 44 were included in the NVP-1703 group and 47 in the placebo group. The dropout rates did not differ between the NVP-1703 and placebo groups (6.8% vs. 8.5%, respectively). Finally, 84 subjects who completed the study without protocol violations were evaluated for primary efficacy outcome (Figure 1). Safety analysis included all the enrolled subjects. Analyses of immunological parameters, TNSS, and RCAT scores were conducted for the subjects who completed the study.

Demographics of the study subjects are summarized in Table 1. The mean age was 33.61 ± 1.23 in the NVP-1703 group and 33.49 ± 1.33 in the placebo group. The proportion of males in the NVP-1703 and placebo group were 24.4 and 44.2%, respectively. The duration of AR was 175.41 ± 14.59 months in the NVP-1703 group and 176.09 ± 13.46 months in the placebo group. Most of the study subjects were sensitized to house dust mites (92.86% in the NVP-1703 group; 95.35% in the placebo group). The baseline TNSS was 8.09 ± 0.29 in the NVP-1703 group and 7.55 ± 0.28 in the placebo group, without significant difference. 

### 3.2. Changes from Baseline in TNSS 

**Figure 2:**
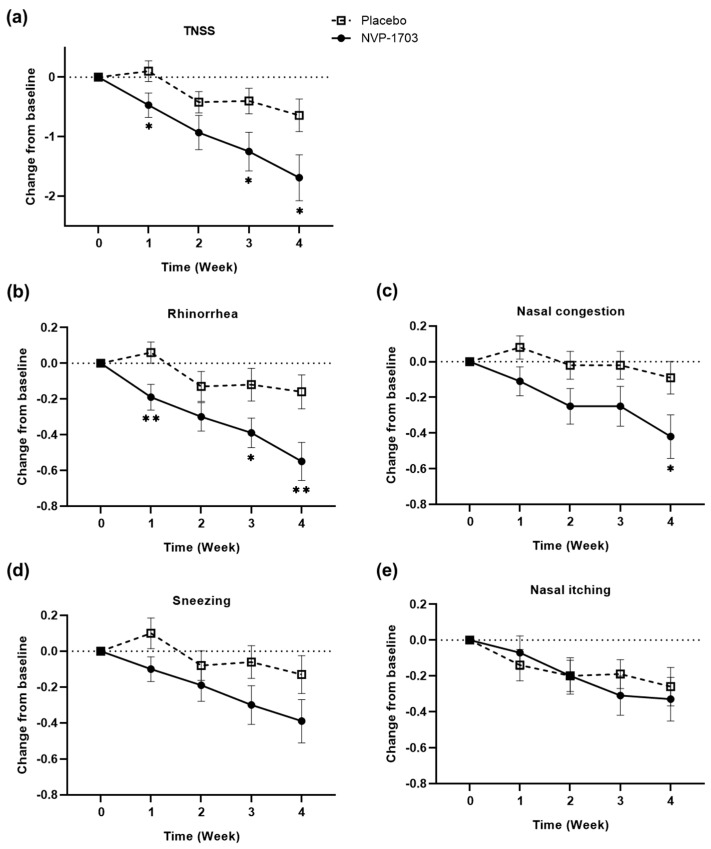
Effect of NVP-1703 on (**a**) TNSS and AR symptoms [(**b**) rhinorrhea, (**c**) nasal congestion; (**d**) sneezing, and (**e**) nasal itching] scores. Scores are shown as weekly mean change in scores from the baseline for total and individual nasal symptoms during the study period. (*) indicates a statistically significant difference between the two groups (* *p* < 0.05, ** *p* < 0.01).

Changes in TNSS and individual nasal symptom scores are summarized in Figure 2 and Table 2. TNSS significantly improved from the baseline to each week (*p* < 0.05) in the NVP-1703 group. Changes in TNSS from baseline to weeks 1, 3, and 4 were −0.47 ± 0.20, −1.25 ± 0.33, and −1.69 ± 0.39, respectively, in the NVP-1703 group, and 0.10 ± 0.17, −0.40 ± 0.21, and −0.64 ± 0.27, respectively, in the placebo group, resulting in significant difference in change between the groups (*p* = 0.033, 0.031, and 0.029, respectively). 

Among individual symptoms, the scores for rhinorrhea were the most markedly reduced from baseline by −0.19 ± 0.07, −0.39 ± 0.08, and −0.55 ± 0.11 at weeks 1, 3, and 4, respectively, in the NVP-1703 group, and 0.06 ± 0.06, −0.12 ± 0.09, and −0.16 ± 0.09, respectively, in the placebo group, showing significant differences between the groups (*p* = 0.007, 0.035, and 0.007, respectively). Furthermore, NVP-1703 treatment significantly reduced the scores for nasal congestion at weeks 2, 3, and 4 compared with those at baseline (*p* = 0.017, *p* = 0.031, and *p* = 0.001, respectively), and a significant difference between the groups was shown at week 4 (−0.42 ± 0.78 in NVP-1703 vs. −0.09 ± 0.60 in placebo, *p* = 0.034). For sneezing and nasal itching scores, there were no significant differences between the groups (*p* > 0.05 each). However, significant improvement from the baseline was observed in the NVP-1703 group at weeks 3 and 4, with changes of −0.30 ± 0.11 and −0.39 ± 0.12, respectively, in sneezing, and −0.31 ± 0.11 and −0.33 ± 0.12, respectively, in itching (*p* < 0.01 each).

### 3.3. Changes from Baseline in RCAT

Total RCAT scores increased from baseline by 1.95 ± 0.58 and 2.00 ± 0.54 in the NVP-1703 and placebo groups, respectively, at week 2 (*p* > 0.05), while at week 4, the scores increased from baseline by 3.83 ± 0.73 and 2.21 ± 0.67, respectively, showing a significant difference in change between the groups (*p* = 0.049). Among the sub-items, watery eyes showed a significant improvement at week 4 (0.80 ± 0.16 with NVP-1703 vs. 0.16 ± 0.19 with placebo, *p* = 0.018) and sleep disturbances tended to improve (0.66 ± 0.13 with NVP-1703 vs. 0.33 ± 0.18 with placebo, *p* = 0.051) (Table 3).

### 3.4. Effect of NVP-1703 on Blood Eosinophil Count and Serum Specific IgE 

There was no significant difference in baseline blood eosinophil counts between the two groups. Although both groups showed significant decreases in blood eosinophil counts at four weeks (*p* < 0.001), there was no significant difference in the changes in counts between the two groups (*p* > 0.05). 

The baseline levels of Dp-specific IgE were 7.61 ± 1.14 and 8.92 ± 1.18 KU/L in the NVP-1703 and placebo groups, respectively (*p* > 0.05), and those of Df-specific IgE were 14.75 ± 2.17 and 16.86 ± 2.20 KU/L, respectively (*p* > 0.05). The serum levels of Df-specific IgE decreased to 12.83 ± 1.78 KU/L at week 4 in the NVP-1703 group (*p* = 0.008), which was significant compared with the change in the placebo group (*p* = 0.033) (Table 4). The serum level of Dp-specific IgE decreased to 7.15 ± 1.04 KU/L at week 4 in the NVP-1703 group (*p* > 0.05 compared with baseline), but there was no significant difference compared with the placebo group. 

### 3.5. Effect of NVP-1703 on Serum Cytokines 

**Figure 3:**
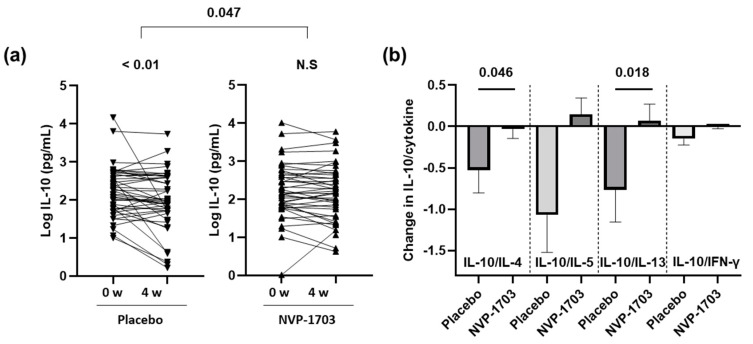
Effects of NVP-1703 on blood levels of IL-10 expression as well as ratios of IL-10 to IL4, IL-5, IL-13, and IFN-γ. (**a**) IL-10 expression at each time point (baseline, 4 weeks) after log transformation; (**b**) change in the ratios of IL-10 to IL-4, IL-5, IL-13, and IFN- γ from baseline at week 4.

The serum levels of IL-4, IL-5, IL-13 and IFN-γ were unchanged after four weeks of treatment with NVP-1703, and there were no statistically significant differences compared with the placebo group. However, the serum level of IL-10 in the NVP-1703 group was maintained, while it was significantly decreased in the placebo group at week 4 (*p* < 0.01), showing significant differences in changes between the two groups (*p* = 0.047). Furthermore, in the NVP-1703 group, the ratio of IL-10 to IL-4 was maintained, with a change of −0.03 ± 0.11 from baseline at week 4, while it decreased by 0.52 ± 0.28 in the placebo group with statistical significance (*p* = 0.046). The ratio of IL-10 to IL-13 was also significantly decreased in the placebo group (0.76 ± 0.39) compared with the NVP-1703 group (0.07 ± 0.20, *p* = 0.018) (Figure 3).

The ratio of IL-10 to IL-5 increased by 0.15 ± 0.20 in the NVP-1703 group (*p* > 0.05), while it decreased by 1.05 ± 0.44 in the placebo group (*p* < 0.05). However, there was no significant difference in change between the groups (*p* = 0.088) (Table 4). 

### 3.6. Effect of NVP-1703 on Urinary LTE_4_ and PGF_2α_

Baseline urinary levels of LTE_4_ were 601.06 ± 32.54 and 546.95 ± 32.18 ng/mmol creatinine in the NVP-1703 and placebo groups, respectively, and those of PGE_2α_ were 77.23 ± 7.66 and 74.19 ± 6.82 ng/mmol creatinine, respectively, without statistical significance between the groups (*p* > 0.05). Although urinary LTE_4_ and PGE_2α_ tended to decrease four weeks after NVP-1703 treatment initiation, compared with the changes in LTE_4_ and PGF_2α_ before and after NVP-1703 treatment, no significant difference in change in urinary levels of LTE_4_ and PGF_2α_ (−22.42 ± 33.47 and −5.38 ± 8.50, respectively) was observed between the groups (*p* > 0.05) (Table 4).

### 3.7. Safety and Compliance

Safety and tolerability (subject-reported symptoms, vital signs, and laboratory tests) were monitored at every visit during the study. No significant difference in any of the vital signs, laboratory tests, or electrocardiograms was observed between the groups. Six adverse events were documented during the study: abdominal pain (one case, 2.1%), muscle strain (one case, 2.1%), nasopharyngitis (one case, 2.1%), and elevation of alanine aminotransferase and aspartate aminotransferase (one case, 2.1%) in the NVP-1703 group, and abdominal pain (one case, 2.1%) and dyspepsia (one case, 2.1%) in the placebo group. No adverse event was regarded to be associated with the study product or placebo. No serious adverse events or dropouts due to adverse events occurred in either the NVP-1703 or placebo group. Compliance with ingestion was 99.87% ± 1.34% and 96.73% ± 1.73% in the NVP-1703 and placebo groups, respectively, without significant difference.

## 4. Discussion

In the present study, NVP-1703 significantly improved TNSS and RCAT scores in subjects with perennial AR. NVP-1703 was especially effective in reducing rhinorrhea, nasal congestion, watery eyes, and sleep disturbances. NVP-1703 also significantly improved allergic immunologic profiles such as Df-specific IgE, IL-10, IL-10/IL-4 ratio, and IL-10/IL-13 ratio. These results suggest that NVP-1703 regulates IL-10, one of the key immunological markers associated with AR, thereby improving symptoms of perennial AR.

Several studies have evaluated the efficacy of probiotics in AR and reported significant improvement in AR symptoms. However, these evaluations of the efficacies of probiotics were limited, because the study subjects continued other medications for AR [7,11,17,18,19,20,21,22,23]. Furthermore, most of the previous studies only used the Quality of Life assessment rather than TNSS to assess efficacy [9,22,23,24,25]. The patient-rated, instantaneous, and reflective TNSS is generally preferred as a primary measure of efficacy at least four weeks in AR trials for drug development [26], because the quality of life assessment may insufficiently or inaccurately evaluate the efficacies of anti-AR drugs and probiotics [10]. In the present study, we independently evaluated the efficacy of NPV-1703 without anti-AR medications and performed both TNSS and quality of life assessments. NVP-1703 showed significant improvement in TNSS as well as RCAT scores. To our knowledge, NVP-1703 is the first probiotic mixture that has been proven to be effective in patients with symptomatic AR without any additional medication or the need for rescue medication. Our results contribute reliable and powerful evidence for the efficacy of probiotics in AR treatment.

Previous clinical trials for AR have evaluated the efficacies of single probiotic strains reported to be effective in preliminary animal studies, including *B. lactis*, *B. longum*, *L. plantatrum*, and *L. paracasei*. However, the effects of a single probiotic strain on AR have not yet been sufficiently evaluated and not consistent between studies. Recently, there have been attempts to mix probiotic strains that have been shown to have anti-allergic effects in animal studies [9,21]. These studies combined different species from within the same genus [9,21]. On this point, our study has some distinct advantages over previous studies. NVP-1703 is a probiotic mixture of *B. longum* IM55 (from the human gut bacteria collection) as a T-cell regulator and *L. plantarum* IM76 (from the fermented food bacteria collection) as a macrophage NF-κB inhibitor. We previously reported that NPV-1703 regulates innate and adaptive immunity in animal and in vitro studies [15,16]. In the present study, NVP-1703 treatment significantly improved TNSS and RCAT scores by increasing levels of IL-10, which corresponds to the anti-allergic effect of *B. longum* IM55 and *L. plantarum* IM76. 

The improvement in clinical outcomes in the NVP-1703 group seems to be related to the improvement in anti-allergic immunologic profiles. NVP-1703 treatment significantly increased serum IL-10 levels. The blood IL-4, IL-5, and IL-13 levels were not significantly changed in the NVP-1703 group; however, the ratios of IL-10/IL-4, IL-10/IL-5, and IL-10/IL-13 were significantly increased in the NVP-1703 group. IL-10 is an important counter-regulatory cytokine required to control allergic inflammation. IL-10-mediated regulation of T-cell responses directly regulates Th2 cells and subsequently decreases IL-4, IL-5, and IL-13 levels in the blood [4]. The administration of NVP-1703 significantly suppressed the expression of IL-4 and IL-5 and differentiation of T-cells into Th2 cells in mice with OVA- and HDMA-induced AR, while the expression of IL-10 and differentiation of T-cells into Treg cells increased [15,16]. These results suggest that NVP-1703 suppresses the expression of Th2 cytokines IL-4, IL-5, and IL-13 by inducing IL-10 expression and Treg cell differentiation.

NVP-1703 treatment also reduced serum Df-specific IgE levels in individuals with AR. IL-10 indirectly facilitates IL-4-induced IgG_4_ production in human B cells, suppresses IgE, and inhibits IgE-induced activation of human mast cells directly in vitro [27,28]. The induction of IL-10 expression suppresses the expression of IgE receptors in mast cells [29]. Therefore, in this study, IL-10 expression induced by NVP-1703 may have suppressed the production of allergen-specific IgE. 

The changes in cytokine profiles and allergen-specific IgE levels with NVP-1703 treatment are similar to those with allergen immunotherapy, which is a disease-modifying, immune-modulatory treatment [5]. Thus, regular ingestion of NVP-1703 may also have long term effects for allergic rhinitis, which requires further studies.

The levels of LTE_4_ and PGF_2α_ in the urine tended to decrease after NVP-1703 treatment compared with the placebo at week 4. Lipid mediators such as cysteinyl LTs and PGD_2_, which are released from eosinophils and mast cells in the early phase of allergic inflammation, play important roles in the pathogenesis of AR [30,31]. These mediators increase venous engorgement and tissue swelling/edema, leading to concomitant rhinorrhea and nasal congestion [30,31]. Their metabolites, urinary LTE_4_ and PGF_2α_, are useful biomarkers of eosinophil or mast cell activation [32]. Leukotriene receptor antagonists, a major type of anti-allergic medicine, exert their action by blocking receptors for cysteinyl LTs [33]. Although our study did not show statistically significant differences, these results suggest that NPV-1703 may be a safe potential anti-allergic medication to treat AR, contributing to an improvement in symptoms, including nasal discharge and nasal congestion. 

A major limitation exists in our study. At first, the duration of probiotic intake was four weeks, which was relatively shorter than the usual eight weeks in previous studies. However, we observed significant symptom improvement at week 4, along with improvement in cytokine profiles, which may be due to the strong effects of the probiotic mixture. In addition, the beneficial effects of the probiotics were observed at least four weeks after continuous administration [34]. To elucidate the mechanisms of their anti-allergic effects, further studies with long-term administration in larger populations will be required. Secondly, we only enrolled the participants with mild to moderate persistent rhinitis who could endure the symptoms without medications. Even though NVP-1703 alone was significantly ameliorate the rhinitis symptoms in our study, we cannot say that NVP-1703 is a better controller than other conventional medications for AR. Further study will be needed to evaluate the comparative or add-on effect of NVP-1703 to other conventional medications. 

## 5. Conclusions

In conclusion, NVP-1703 improves AR symptoms, which results from IL-10 expression in Treg cells. NVP-1703 may reduce the secretion of IL-4, IL-5, IL-13, allergen-specific IgE, PGD_2_, and cysteinyl LTs by suppressing the activation of mast cells, eosinophils, Th2 cells, and B cells through the induction of Treg cells. However, further studies are required to elucidate this mechanism.

## Figures and Tables

**Table 1:** Baseline characteristics of the study subjects.

Characteristics	NVP-1703 (*n* = 41)	Placebo (*n* = 43)	*p*-Value
Male [*n* (%)]	10 (24.39)	19 (44.19)	0.057
Age, years [mean (SEM)]	33.61 (1.23)	33.49 (1.33)	0.947
Duration of allergic rhinitis, months [mean (SEM)]	175.41 (14.59)	176.09 (13.46)	0.923
Family history of allergic diseases [*n* (%)]	33 (80.49)	33 (76.74)	0.676
Positivity of skin prick test [*n* (%)]			
*D. pteronyssinus*	38 (92.86)	39 (90.70)	1.000
*D. farinae*	38 (92.68)	41 (95.35)	0.672
Mold 1	1 (2.44)	2 (4.65)	1.000
Mold 2	0	1 (2.33)	1.000
Cat	7 (17.07)	5 (11.63)	0.688
Dog	6 (14.63)	5 (11.63)	0.933
Cockroach	5 (12.20)	4 (9.30)	0.735
Tree pollen mixture	4 (9.76)	9 (20.93)	0.265
Birch	7 (17.07)	11 (25.58)	0.494
Grass pollen mixture	3 (7.32)	4 (9.30)	1.000
Ragweed	4 (9.76)	3 (6.98)	0.710
Mugwort	7 (17.07)	5 (11.63)	0.688
Symptom score [mean (SEM)]			
TNSS	8.09 (0.29)	7.55 (0.28)	0.174
Rhinorrhea	2.29 (0.07)	2.03 (0.09)	0.031
Nasal congestion	2.12 (0.12)	1.95 (0.12)	0.292
Sneezing	1.88 (0.10)	1.68 (0.12)	0.203
Itching	1.81 (0.14)	1.89 (0.12)	0.661

Data are expressed as mean (SEM). TNSS, total nasal symptom score; Mold 1, *Alternaria alternata*, *Chaetomium globosum*, *Cladosporium*, *Helminthosporium sativum*; Mold 2, *Aspergillus fumigatus*, *Aspergillus niger*.

**Table 2:** Changes in TNSS and individual symptom scores during the study period.

Parameter	Group	Symptom Score				
		0 w	1 w	2 w	3 w	4 w
TNSS	NVP-1703	8.09 (0.29)	7.62 (0.29) *	7.16 (0.35) ***	6.84 (0.37) ***	6.40 (0.41) ***
	Placebo	7.55 (0.28)	7.65 (0.31)	7.13 (0.33) *	7.15 (0.34)	6.90 (0.36) *
	*p*-Value ^†^		0.033	0.135	0.031	0.029
Rhinorrhea	NVP-1703	2.29 (0.07)	2.09 (0.09) *	1.99 (0.09) ***	1.90 (0.09) ***	1.73 (0.11) ***
	Placebo	2.03 (0.09)	2.10 (0.11)	1.90 (0.11)	1.91 (0.11)	1.87 (0.11)
	*p*-Value ^†^		0.007	0.149	0.035	0.007
Nasal congestion	NVP-1703	2.12 (0.12)	2.02 (0.11)	1.87 (0.12) *	1.87 (0.12) *	1.71 (0.12) **
	Placebo	1.95 (0.12)	2.03 (0.12)	1.93 (0.11)	1.93 (0.11)	1.85 (0.12)
	*p*-Value ^†^		0.077	0.070	0.097	0.034
Sneezing	NVP-1703	1.88 (0.10)	1.77 (0.09)	1.69 (0.10) *	1.57 (0.12) **	1.49 (0.13) **
	Placebo	1.68 (0.12)	1.78 (0.10)	1.60 (0.11)	1.62 (0.12)	1.55 (0.12)
	*p*-Value ^†^		0.069	0.374	0.087	0.103
Itching	NVP-1703	1.81 (0.14)	1.74 (0.11)	1.61 (0.12)	1.50 (0.13) **	1.47 (0.13) **
	Placebo	1.89 (0.12)	1.75 (0.11)	1.69 (0.11) *	1.69 (0.11) *	1.63 (0.12) *
	*p*-Value ^†^		0.597	0.955	0.394	0.641

Data are expressed as mean (SEM) for TNSS and individual symptoms. ^†^ *p-*value for change in symptom score from baseline between NVP-1703 and placebo groups. * *p* < 0.05, ** *p* < 0.01, *** *p* < 0.001 for change in symptom score from baseline within groups. TNSS, total nasal symptom score.

**Table 3:** Changes in RCAT and individual symptom scores during the study period.

Parameter	Group	Symptom Score		
		0 w	2 w	4 w
Total	NVP-1703	15.44 (0.50)	17.39 (0.62) **	19.27 (0.70) ***
	Placebo	15.26 (0.44)	17.26 (0.51) ***	17.47 (0.56) **
	*p*-Value ^†^		0.957	0.049
Nasal congestion	NVP-1703	1.95 (0.14)	2.20 (0.15)	2.63 (0.16) ***
	Placebo	2.00 (0.11)	2.33 (0.12) **	2.35 (0.13) **
	*p*-Value ^†^		0.523	0.086
Sneezing	NVP-1703	2.27 (0.12)	2.56 (0.12) **	2.83 (0.15) **
	Placebo	2.19 (0.13)	2.63 (0.13) **	2.51 (0.14)
	*p*-Value ^†^		0.483	0.143
Watery eyes	NVP-1703	2.68 (0.15)	3.05 (0.14) *	3.49 (0.16) ***
	Placebo	2.88 (0.15)	3.12 (0.16)	3.05 (0.17)
	*p*-Value ^†^		0.933	0.018
Interference of Sleep	NVP-1703	3.07 (0.12)	3.29 (0.16)	3.73 (0.14) ***
	Placebo	3.02 (0.14)	3.26 (0.12)	3.35 (0.14)
	*p*-Value ^†^		0.960	0.051
Daily activities	NVP-1703	3.15 (0.15)	3.39 (0.15)	3.59 (0.18) *
	Placebo	2.91 (0.12)	3.28 (0.13) *	3.42 (0.12) **
	*p*-Value ^†^		0.955	0.679
Degree of rhinitis control	NVP-1703	2.32 (0.10)	2.90 (0.12) ***	3.00 (0.13) ***
	Placebo	2.26 (0.09)	2.65 (0.12) *	2.79 (0.12) **
	*p*-Value ^†^		0.161	0.237

Data are expressed as mean (SEM) of symptom score. ^†^ *p*-value for change in symptom score from baseline between NVP-1703 and placebo groups. * *p* < 0.05, ** *p* < 0.01, *** *p* < 0.001 for change in symptom score from baseline within groups. RCAT, rhinitis control assessment test.

**Table 4:** Changes in immunological parameters.

Parameter	NVP-1703		Placebo		
	0 w	4 w	0 w	4 w	*p*-Value ^†^
Blood eosinophil count (/μL)	175.42 (16.21)	96.82 (25.24) ***	178.24 (17.14)	56.61 (14.35) ***	0.116
*D. pteronyssinus*-specific IgE (KU/L)	7.61 (1.14)	7.15 (1.04)	8.92 (1.18)	8.93 (1.22)	0.331
*D. farinae*-specific IgE (KU/L)	14.75 (2.17)	12.83 (1.78) **	16.86 (2.20)	17.19 (2.35)	0.033
IL-4 (log pg/mL)	2.29 (0.12)	2.31 (0.12)	2.28 (0.12)	2.28 (0.12)	0.940
IL-5 (log pg/mL)	1.65 (0.12)	1.59 (0.14)	1.36 (0.12)	1.34 (0.11)	0.605
IL-10 (log pg/mL)	2.22 (0.11)	2.16 (0.11)	2.23 (0.09)	1.97 (0.11)^**^	0.047
IL-13 (log pg/mL)	1.49 (0.10)	1.47 (0.12)	1.55 (0.10)	1.51 (0.10)	0.936
IFN-γ (log pg/mL)	2.99 (0.08)	2.98 (0.09)	2.85 (0.08)	2.84 (0.08)	0.488
IL-10/IL-4	1.21 (0.15)	1.17 (0.16)	1.42 (0.28)	0.90 (0.09)	0.046
IL-10/IL-5	2.15 (0.24)	2.27 (0.27)	3.30 (0.47)	2.27 (0.22) *	0.088
IL-10/IL-13	2.73 (0.42)	2.77 (0.44)	2.71 (0.41)	1.95 (0.17)	0.018
IL-10/IFN-γ	0.53 (0.05)	0.52 (0.05)	0.64 (0.08)	0.49 (0.04)	0.177
Urinary LTE_4_ (ng/mmol Cr)	601.06 (32.53)	585.78 (31.24)	546.95 (32.18)	541.23 (32.31)	0.701
Urinary PGF_2α_ (ng/mmol Cr)	77.23 (7.66)	69.90 (7.90)	74.19 (6.82)	72.13 (6.27)	0.809

Data are expressed as mean (SEM). Cytokine levels were analyzed after logarithmic transformation. † *p*-value for change from baseline at 4 weeks between NVP-1703 and placebo groups. * *p* < 0.05, ** *p* < 0.01, *** *p* < 0.001 for change from baseline at 4 weeks within groups.
